# Delayed reversibility of complete atrioventricular block: cardio-biliary reflex after alcohol septal ablation in a patient with hypertrophic obstructive cardiomyopathy

**DOI:** 10.1186/s12872-021-02165-5

**Published:** 2021-08-03

**Authors:** Shu Fang, Lan Gao, Fan Yang, Yan-jun Gong

**Affiliations:** Department of Cardiology, Peking University First Hospital, No.8 Xishiku Street, Xicheng District, Beijing, 100034 China

**Keywords:** Cholecystitis, Cholangitis, Complete atrioventricular block, Cardio-biliary reflex, Alcohol septal ablation

## Abstract

**Background:**

Complete atrioventricular block (AVB) is a life-threatening condition that usually occurs in elderly people with organic heart disease. We herein describe a rare case of complete AVB in a young man with hypertrophic obstructive cardiomyopathy (HOCM) complicated by cholecystitis and cholangitis. Both cardio-biliary reflex and alcohol septal ablation (ASA) can cause conduction block, but the latter is often irreversible. However, their simultaneous occurrence in a patient has not been reported.

**Case presentation:**

A 31-year-old man presented with acute cholecystitis and cholangitis and complete AVB, which had been diagnosed at a local hospital on the third day after onset. On the fourth day, he was transferred to the emergency department of our hospital because of persistent complete AVB, although his abdominal pain had been partially relieved. An echocardiogram showed a remarkably elevated left ventricular outflow tract (LVOT) gradient (105.2 mmHg) despite the performance of ASA 9 years previously. The abdominal pain gradually disappeared, and normal sinus rhythm was completely recovered 11 days after onset. We determined that cardio-biliary reflex was the cause of the AVB because of the absence of other common causes. Finally, the patient underwent implantation of a permanent pacemaker to reduce the LVOT obstruction and avoid the risk of AVB recurrence.

**Conclusions:**

Cholecystitis is a rare cause of complete AVB, which is a difficult differential diagnosis when complicated by HOCM after ASA. Clinicians should be alert to the possibility of cholecystitis in patients with abdominal pain and an unknown cause of bradycardia, complete AVB, or even sinus arrest.

**Supplementary Information:**

The online version contains supplementary material available at 10.1186/s12872-021-02165-5.

## Background

Complete atrioventricular block (AVB) is characterized as independent atrial and ventricular activity due to anatomic or functional disorders of the conduction system. Complete AVB may have physiologic, pathophysiologic, or iatrogenic etiologies, some of which are reversible [1]. Acute cholecystitis is a common surgical disease that is associated with arrhythmias or ST-T wave changes [2] through increased vagal tone. The latter, known as the cardio-biliary reflex, is self-restoring on an electrocardiogram (ECG) [3]. Alcohol septal ablation (ASA) was introduced in 1994 as an effective treatment option and an alternative to surgical myectomy for patients with hypertrophic obstructive cardiomyopathy (HOCM), helping to reduce left ventricular outflow tract obstruction (LVOTO) and associated symptoms. ASA-induced myocardial necrosis occurs in the basal septum, which closed to the cardiac conduction system and likely to cause conduction block [4].

This is the first report of 9 days of complete AVB complicated by biliary tract infection after ASA. In this mixed situation, a thorough clinical history and examination were important for accurate diagnosis and treatment.

## Case presentation

**Fig. 1:**
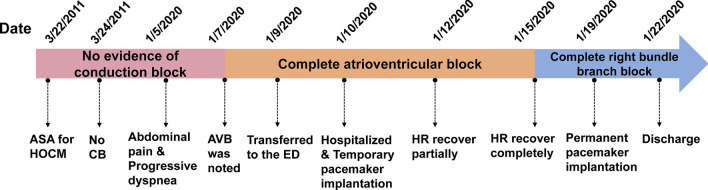
Timeline of events. *ASA* alcohol septal ablation, *HOCM* hypertrophic obstructive cardiomyopathy, *CB* conduction block, *AVB* atrioventricular block, *ED* emergency department, *HR* heart rhythm

**Fig. 2:**
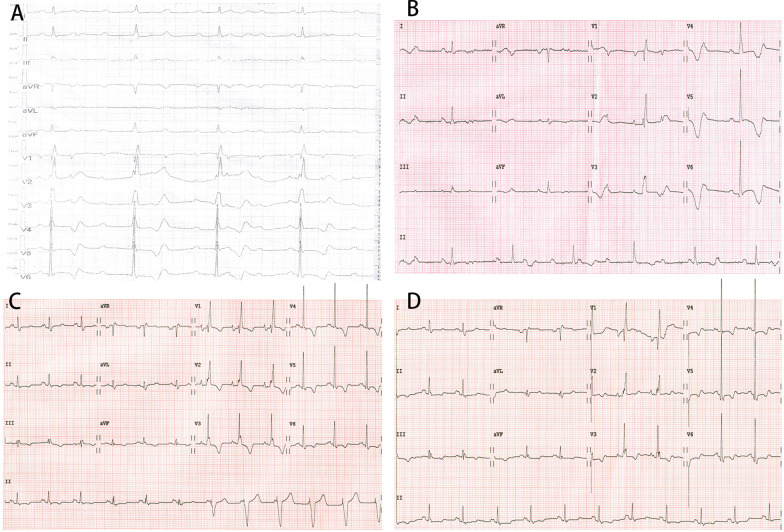
Dynamic electrocardiogram changes on patient presentation. **A** Complete atrioventricular block (AVB) on the third day after onset in the local hospital. **B** Transferred to the emergency department of our hospital on the fifth day after onset. **C** Sinus rhythm partially passed down on eighth day after onset. **D** Complete right bundle branch block on 11th day after onset when AVB had disappeared

A 31-year-old man presented with acute postprandial abdominal pain, vomiting, and progressive dyspnea until he was unable to walk on 5 January 2020 (Fig. 1). The patient was diagnosed with biliary tract infection and complete AVB (Fig. 2A) at a local hospital. Although his abdominal pain was partially relieved after anti-infective therapy, the complete AVB and dyspnea remained. He was transferred to the emergency department of our hospital on the fourth day after onset. He was afebrile with stable hemodynamics and showed no hypoxemia. He was able to lie supine without jugular vein distention and had rales with diminished breath sounds at both lung bases. A cardiac murmur was strongest at the left margin of the sternum between the third and fourth intercostal spaces; it was a grade III/VI total systolic murmur radiating to the periphery, armpit, and back. Tenderness was present in the upper abdomen with rebound pain and muscle tension, and Murphy’s sign was negative. The patient was diagnosed with HOCM in 2011 (Additional file 1: Figure S1). ASA was performed in 2011 because of recurrent syncope with a remarkably high left ventricular outflow tract (LVOT) gradient (125 mmHg) after the Valsalva maneuver. The patient was a deliveryman without restrictions for performing heavy manual labor after ASA. He denied taking any medications.

**Fig. 3:**
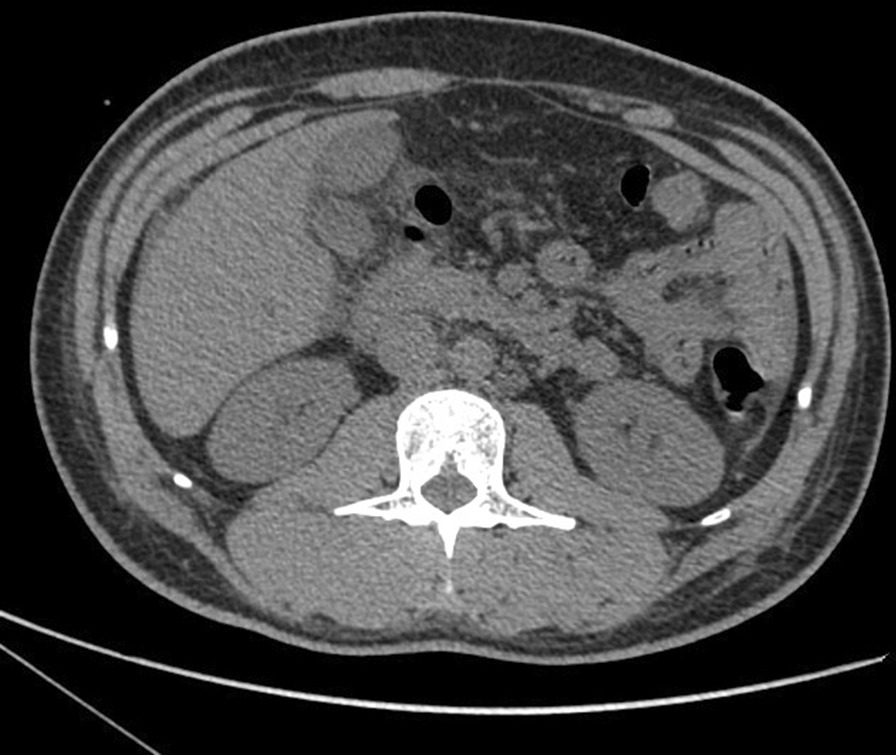
Abdominal computed tomography. The gallbladder wall was edematous and thickened, and no gallstone was observed

The patient was then sent to the cardiac care unit. Laboratory tests showed a white blood cell count of 10.01 × 10^9^/L (reference range, 3.5–9.5 × 10^9^/L), neutrophil percentage of 65.7%, alanine aminotransferase level of 257 IU/L (reference range, 9–50 IU/L), aspartate aminotransferase level of 110 IU/L (reference range, 15–40 IU/L), total bilirubin level of 54 µmol/L (reference range, 1.7–20 µmol/L), brain natriuretic peptide level of 2717 pg/mL (reference range, < 100 pg/mL), C-reactive protein level of 19 mg/L (reference range, < 5 mg/L), and procalcitonin level of 0.18 ng/mL (reference range, < 0.05 ng/mL). Serum electrolytes, troponin I, thyroid hormone, multiple blood cultures, antinuclear antibody, anti-dsDNA, complement C3/C4, rheumatoid factor, and anti-streptolysin O were all normal or negative. ECG showed complete AVB with junctional escape at 40 beats/min (Fig. 2B). Chest radiographs showed no lung lesions (Additional file 2: Figure S2). Abdominal computed tomography showed cholecystitis and cholangitis with peripheral exudation without stones (Fig. 3).

**Fig. 4:**
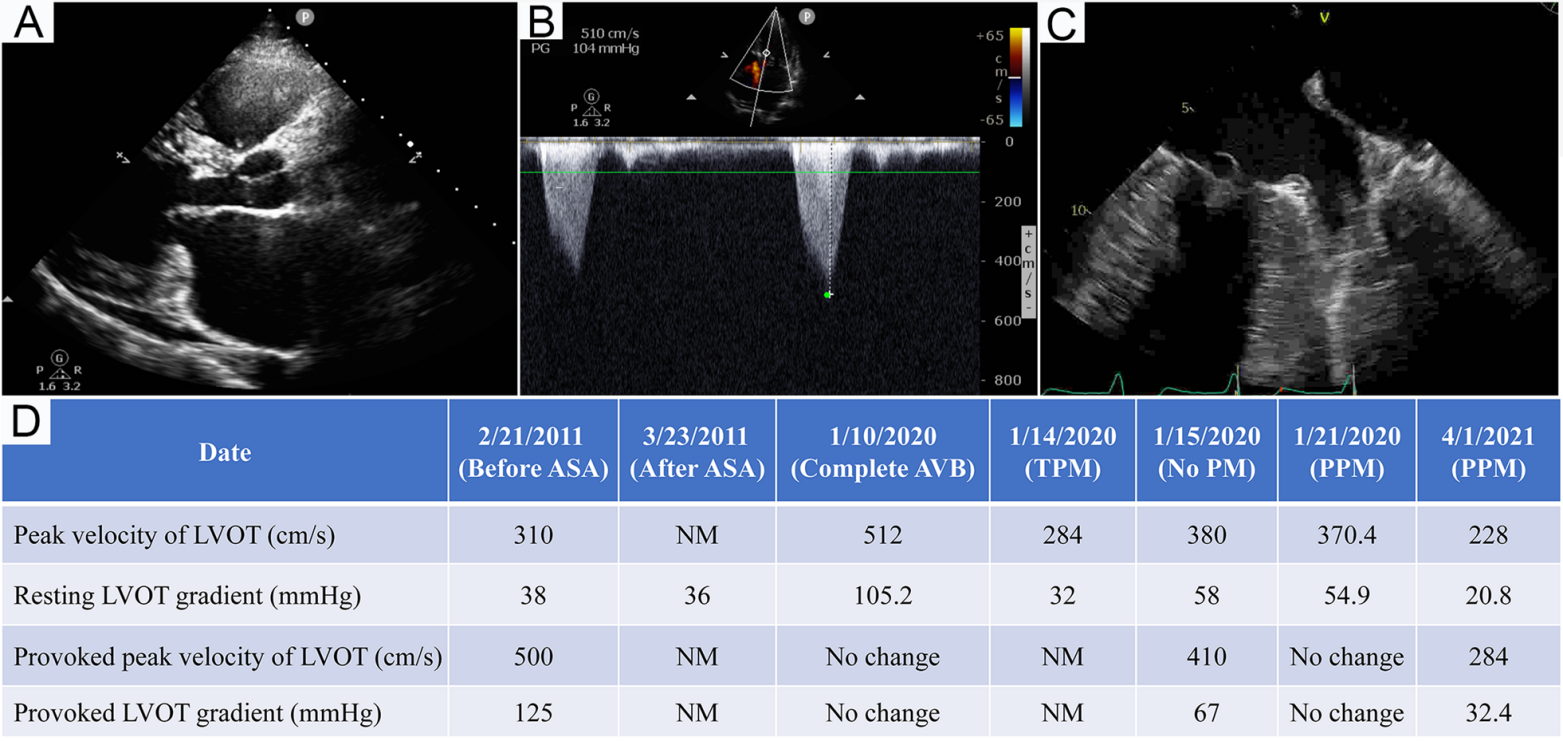
Echocardiogram. **A** Parasternal long-axis view on admission. **B** Doppler ultrasonic spectrum of LVOT on admission. **C** Transesophageal echocardiograph showed mitral valve prolapse and no vegetation. **D** Dynamic change of LVOT gradient and peak velocity. This patient underwent alcohol septal ablation on March 22, 2011 and permanent pacemaker on January 19, 2020. *LVOT* left ventricular outflow tract, *ASA* alcohol septal ablation, *NM* not measured, *AVB* atrioventricular block, *TPM* temporary pacemaker, *PM* pacemaker, *PPM* permanent pacemaker

Given the concern for heart failure and the previously auscultated cardiac murmur, the patient underwent echocardiographic examination. This showed mitral valve prolapse and a suspected mass, a left ventricular ejection fraction of 69.7%, and LVOTO with a gradient of 105.2 mmHg without change after the Valsalva maneuver (Fig. 4A, B). Subsequent transesophageal echocardiography revealed no valvular vegetation or mass (Fig. 4C). The patient underwent implantation of a temporary pacemaker on admission and was treated with meropenem, hepatoprotective drugs, and nutritional support.

The patient reported no abdominal pain when palpated on day 7 after onset. Unexpectedly, on day 8, we found that sinus rhythm could be partially passed down when attempting to reduce the pacing frequency (Fig. 2C). On the 11th day, the complete AVB disappeared, and the ECG showed complete right bundle branch block (Fig. 2D). During hospitalization, the peak velocity LVOT and LVOT gradient at rest and when provoked were monitored dynamically (Fig. 4D), and the results suggested that the LVOTO was still present even when normal rhythm was restored, either at rest or when provoked, and the pacemaker effectively reduced the LVOTO. Finally, the patient underwent permanent pacemaker implantation to reduce the LVOTO and prevent recurrence of AVB.

The patient was treated with a β-blocker and dual chamber pacing. After 18 months of follow-up, his New York Heart Association classification decreased from III to I without episodes of abnormal symptoms.

## Discussion and conclusions

The causes of complete AVB are complex and various. We excluded myocardial infarction and myocarditis in this case because of the negative troponin I. Infective endocarditis could also be excluded because no valvular vegetations were present on transesophageal echocardiography. The lack of multisystem involvement made infiltrative cardiomyopathy an exception. Hyperkalemia, hyperthyroidism, and hypothyroidism could be easily ruled out based on the negative laboratory tests. In addition, AVB caused by progressive cardiac conduction system disease and cardiomyopathy are often irreversible.

The first differential diagnosis that we considered in this patient was a complication of the ASA procedure. Atrioventricular conduction disturbances following ASA are mainly seen in elderly patients, and pacemaker implantation is needed in only 5% of young patients [5, 6]. Patients with left bundle branch block, a P-R interval of > 200 ms, a greater pressure gradient during exercise before ablation, and advanced age are more likely to develop AVB after ASA [7, 8]. Complete AVB after ASA is usually seen within the first 24 h [9]. In one study, the incidence of late complete AVB was 8.9% more than 48 h after ASA and only 3.6% from 4 days to 3 years after ASA; no cases of complete AVB occurred after 3 years [8]. Late complete AVB may be explained by myocardial scarring and fibrosis after ASA [8, 10], and these changes are progressive and unlikely recoverable. Our patient had few risk factors for AVB, and no AVB was found within 48 h after ablation (Additional file 3: Figure S3). Complete AVB can aggravate LVOTO, which in turn leads to acute heart failure [11]. However, our patient had no typical symptoms of heart failure or episodes of syncope in daily life with heavy manual labor after ASA until the onset of acute biliary tract infection. Therefore, ASA was excluded as the cause of AVB, and the cardio-biliary reflex was determined to be the rare cause of the reversible AVB.

The cardio-biliary reflex is triggered by increased tension in the gallbladder via autonomic vagal innervations in patient with cholecystitis, regardless of the presence of pain or calculi [12, 13]. Bradycardia is one of the clinical presentations and is termed “Cope’s sign”; this phenomenon was first documented in 1971 by O'Reilly and Krauthamer [3]. The cardio-biliary reflex also mimics the special ECG changes of acute coronary syndrome, such as ST-segment elevation and T-wave inversion, which are identified and treated by atropine [2, 13, 14]. The cardio-biliary reflex is often a diagnosis of exclusion. Because of the lack of evidence of other diseases in this case, we speculated the cardio-biliary reflex was the predominant cause of the complete AVB. As shown in Table 1, only five such cases of conduction block have been reported, and the longest duration was about 48 h [12, 15–18]. Similar to the case reported by Lau et al. [12], our patient had persistent AVB despite disappearance of his abdominal pain, indicating that the inflammatory response may have resulted in prolonged activation of the cardio-biliary reflex although his inflammatory markers were slightly raised. Notably, complete right bundle branch block may have been involved in the unusually long duration of complete AVB, which was not mentioned in any previous cases.

**Table 1:** Review of case report on cardio-biliary reflex presented with bradycardia (not include arrhythmias during cholecystectomy)

Year	Age	Sex	Diagnosis	Comorbidity	Electrocardiogram	Inflammatory marks	Treatment	Recover time to normal rhythm	Flow up	References
1967	56	M	Calculous cholecystitis	Healthy	Sinus bradycardia with PVC, inverted T waves in V3 to V6	NA	Atropine, cholecystectomy	After injection of atropine	NA	[2]
1969	81	M	Cholecystitis and cholelithiasis	NA	Sinus bradycardia	Slightly raised WBC	Cholecystectomy	After cholecystectomy	NA	[3]
1970	56	M	Acute calculous cholecystitis	NA	Sinus bradycardia	WBC 11 × 10^9^ /L	Atropine, antibiotics	36–48 h with abdominal pain disappeared	NA	[3]
1999	67	F	Severe chronic cholecystitis with perforation	Thoracic aortic aneurysm, aortoesophageal fistula	High-grade AVB and asystole	NA	Percutaneous drainage, cholecystectomy	After percutaneous drainage	Died from UGTB 1 day after operation	[17]
2009	48	M	Acute cholecystitis	Healthy	Complete AVB and second-degree heart block	NA	Cholecystectomy	9 s	1 year	[16]
2011	35	M	Acute calculous cholecystitis	Healthy	Sinus bradycardia and ventricular rhythm	NA	NA	20 min	NA	[14]
2015	70	M	Acalculous cholecystitis	Healthy	Sinus bradycardia with 4.44 s pause	WBC 12 × 10^9^ /L (4–11 × 10^9^ /L)	Cholecystectomy	NA	NA	[12]
2018	78	M	Cholelithias	Hypertension, dyslipidemia, CAD	AVB	CRP 2,71 mg/dL (0.08–0.8)	Antibiotic, analgesics	1 h	Planned cholecystectomy	[15]
2020	28	M	Acute calculous cholecystitis	NA	Complete AVB	Mildly elevated	Cholecystectomy, TPM implantation	At least 10 h	NA	[18]
-	31	M	Acute cholecystitis and cholangitis without stones	HOCM	Complete AVB	WBC 10.01 × 10^9^ /L CRP 19.0 mg/L, PCT 0.18 ng/mL	Antibiotic, PM implantation	At least 9 days	18 months	This case

*M* male, *F* female, *NA* not available, *HOCM* hypertrophic obstructive cardiomyopathy, *CAD* coronary artery disease, *AVB* atrioventricular block, *PVC* premature ventricular contractions, *WBC* white blood cell, *CRP* C-reactive protein, *PCT* procalcitonin, *UGTB* upper gastrointestinal tract bleeding, *TPM* temporary pacemaker, *PM* pacemaker

According to the European Society of Cardiology guidelines on cardiac pacing and cardiac resynchronization therapy, permanent pacemaker implantation can be completely avoided if the conduction block is curable and self-recoverable [19]. Pacemaker therapy is not as effective as surgery or alcohol septal reduction in reducing the gradient; older patients are more likely to benefit from these procedures, and septal myectomy is recommended if mitral valve repair is required for treatment of mitral valve prolapse [11]. Our patient refused cholecystectomy and septal myectomy plus mitral valve prolapse repair surgery. After a multidisciplinary discussion, permanent pacemaker implantation was recommended based on the following three considerations: first, the cholecystitis may recur, which may cause recurrence of the AVB; second, a permanent pacemaker may reduce the LVOTO; and third, a pacemaker may provide security for the use of a β-blocker.

This case revealed a rare etiology and the longest duration (at least 9 days) of complete AVB complicated by acute biliary tract infection and HOCM after ASA. Cholecystitis causes multiple ECG abnormalities, and close monitoring and management are necessary to avoid misdiagnosis and possible complications. Further research is needed to elucidate the mechanism of the cardio-biliary reflex.

## Supplementary Information

Additional file 1: Figure S1. Cardiac magnetic resonance imaging.

Additional file 2: Figure S2. Chest radiograph.

Additional file 3: Figure S3. Electrocardiogram in 2011.

## Data Availability

Data are available from the corresponding author upon reasonable request.

## Abbreviations

ASA: Alcohol septal ablation
HOCM: Hypertrophic obstructive cardiomyopathy
AVB: Atrioventricular block
ECG: Electrocardiogram
LVOT: Left ventricular outflow tract
LVOTO: Left ventricular outflow tract obstruction
