# Supplementary material for: Delayed reversibility of complete atrioventricular block: cardio-biliary reflex after alcohol septal ablation in a patient with hypertrophic obstructive cardiomyopathy
Source: BMC Cardiovasc Disord. 2021 Aug 3;21:372. doi: 10.1186/s12872-021-02165-5 (PMC8330103; doi:10.1186/s12872-021-02165-5)
Supplement: Supplementary file 1 — Cardiac magnetic resonance imaging. [file 12872_2021_2165_MOESM1_ESM.docx]

**
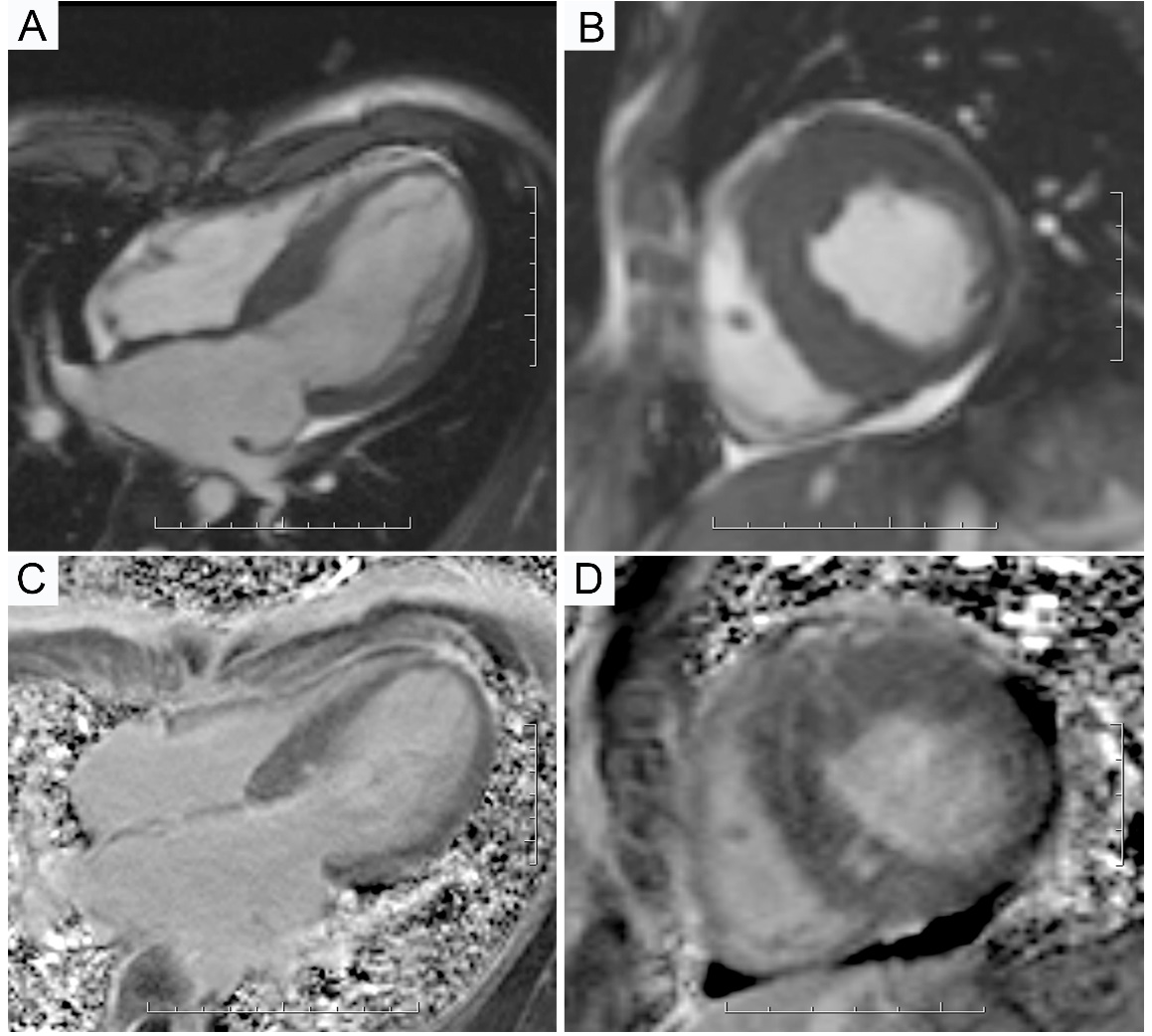
**

**Figure S1**. **Cardiac magnetic resonance imaging**. Gadolinium‐enhanced scan showed significant thickening of interventricular septum (A, B); delayed enhancement imaging showed focal enhancement in the basal segment of the interventricular septum (C, D).
