# Supplementary material for: Delayed reversibility of complete atrioventricular block: cardio-biliary reflex after alcohol septal ablation in a patient with hypertrophic obstructive cardiomyopathy
Source: BMC Cardiovasc Disord. 2021 Aug 3;21:372. doi: 10.1186/s12872-021-02165-5 (PMC8330103; doi:10.1186/s12872-021-02165-5)
Supplement: Supplementary file 2 — Chest radiograph. [file 12872_2021_2165_MOESM2_ESM.docx]

**
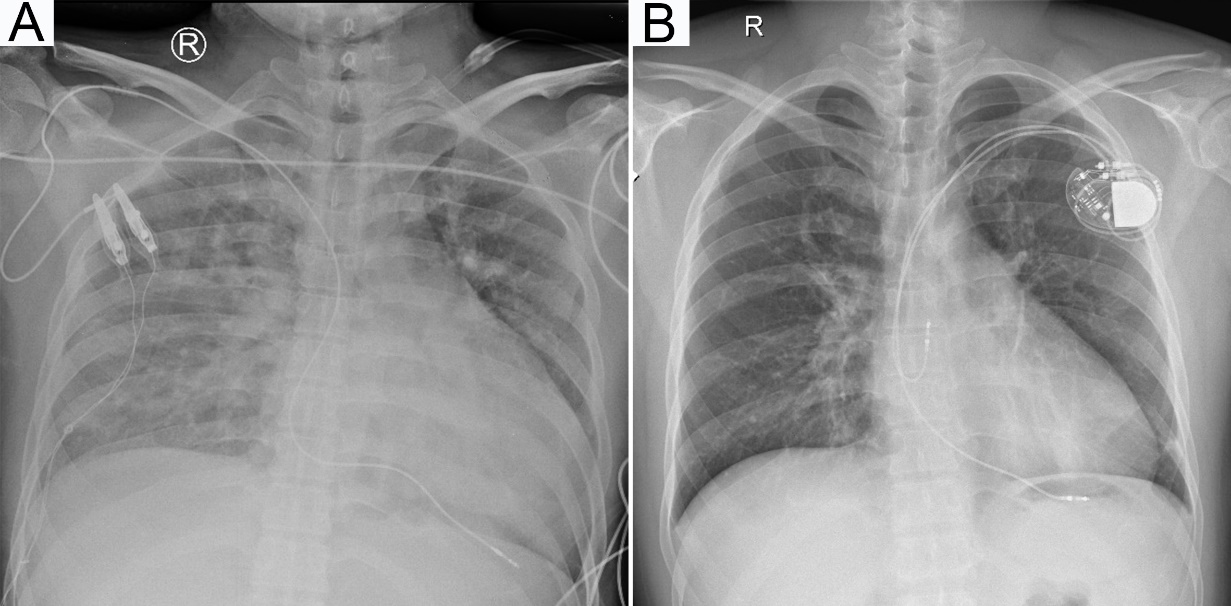
**

**Figure S2 Chest radiograph.** Chest radiograph showed evident signs of heart failure with multiple patch fuzzy shadows on both lungs (A). Cardiac silhouette enlargement without lung field lesions at 3 months after discharge (B).
