# Supplementary material for: Delayed reversibility of complete atrioventricular block: cardio-biliary reflex after alcohol septal ablation in a patient with hypertrophic obstructive cardiomyopathy
Source: BMC Cardiovasc Disord. 2021 Aug 3;21:372. doi: 10.1186/s12872-021-02165-5 (PMC8330103; doi:10.1186/s12872-021-02165-5)
Supplement: Supplementary file 3 — Electrocardiogram in 2011. [file 12872_2021_2165_MOESM3_ESM.docx]

**
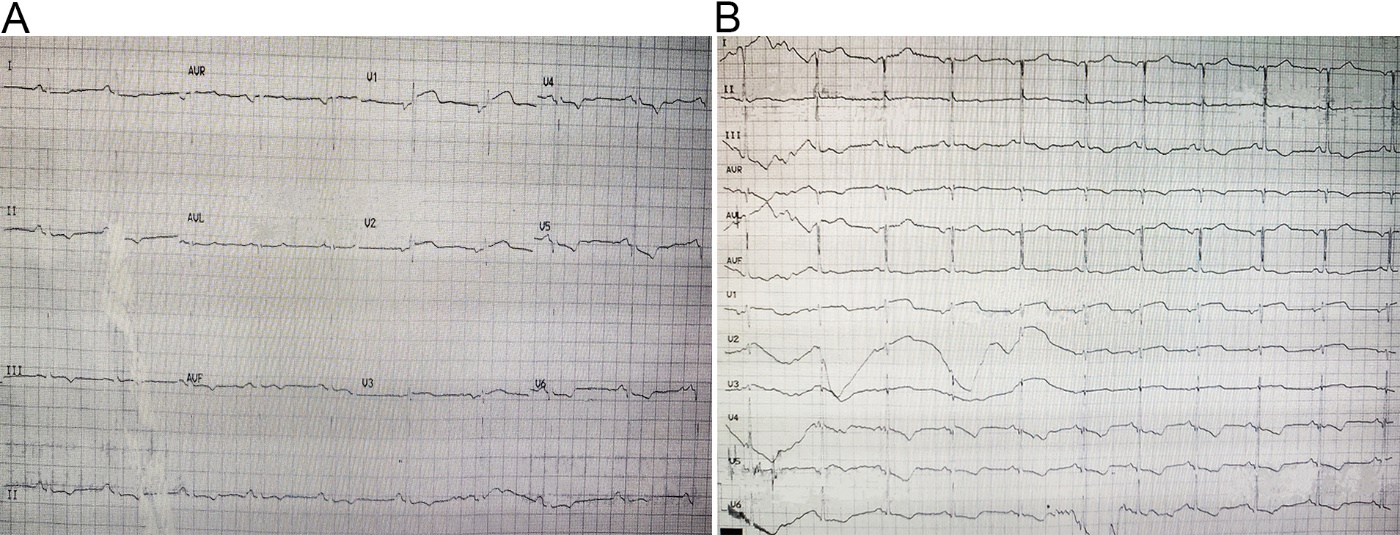
**

**Figure S3 Electrocardiogram in 2011.** Before the alcohol septal ablation (A) and after alcohol septal ablation within 48 hours (B).
